# The risk of epilepsy after neonatal seizures

**DOI:** 10.1111/dmcn.16255

**Published:** 2025-02-19

**Authors:** Jeanette Tinggaard, Signe V. Pedersen, Mads L. Larsen, Andreas K. Jensen, Gorm Greisen, Bo M. Hansen, Christina E. Hoei‐Hansen

**Affiliations:** ^1^ Department of Paediatrics and Adolescent Medicine Copenhagen University Hospital, Rigshospitalet Copenhagen Denmark; ^2^ Department of Obstetrics Copenhagen University Hospital, Rigshospitalet Copenhagen Denmark; ^3^ Department of Public Health, Section of Biostatistics University of Copenhagen Copenhagen Denmark; ^4^ Department of Clinical Medicine University of Copenhagen Copenhagen Denmark; ^5^ Department of Neonatology Copenhagen University Hospital, Rigshospitalet Copenhagen Denmark; ^6^ Department of Pediatrics and Adolescent Medicine Copenhagen University Hospital Hilleroed Denmark

## Abstract

**Aim:**

To estimate the cumulative risk of epilepsy after neonatal seizures and identify subpopulations at increased risk.

**Method:**

This was a nationwide register‐based cohort study including all children born in Denmark between 1997 and 2018. The cumulative risk of epilepsy in children with and without neonatal seizures was compared. Furthermore, neonatal seizures were stratified according to aetiology.

**Results:**

We followed 1 294 377 children and identified 1998 neonatal survivors with neonatal seizures. The cumulative risk of epilepsy was 20.4% (95% confidence interval [CI] = 18.5–22.3) among children with neonatal seizures, compared to 1.15% (95% CI = 1.12–1.18) among children without. Epilepsy was diagnosed before 1 year of age in 11.4% of children with neonatal seizures, in an additional 4.5% between 1 year and 5 years, 3.1% between 5 years and 10 years, and 1.4% between 10 years and 22 years. The aetiologies of neonatal cerebral infarction, haemorrhage, or malformations (adjusted hazard ratio = 2.49, 95% CI = 1.98–3.14) and low Apgar score (1.49, 95% CI = 1.12–1.98) were associated with the highest risk of epilepsy, compared to children with seizures of unknown aetiology.

**Interpretation:**

Epilepsy after neonatal seizures is common and remains a substantial risk throughout childhood. Aetiological risk factors are identifiable and relevant when planning appropriate information for parents and follow‐up.

## Graphical abstract

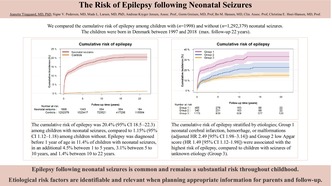

Plain language summary: https://onlinelibrary.wiley.com/doi/10.1111/dmcn.16288

**This original article is commented by Nagarajan on pages** 1112–1113 **of this issue.**

**Abbreviation**
ILAE: International League Against Epilepsy

**What this study adds**

- After neonatal seizures, the risk of epilepsy is persistently increased throughout adolescence.
- Approximately one in five among neonatal survivors with neonatal seizures develop epilepsy.
- Structural brain abnormalities are associated with the greatest risk of epilepsy.
- The risk of febrile seizures alone is also increased after neonatal seizures.

Neonatal seizures are one of the most frequent acute neurological conditions among infants admitted to neonatal care units, with a reported incidence of 1 to 5 cases per 1000 live births.^1^, ^2^, ^3^, ^4^, ^5^, ^6^ Neonatal seizures are often caused by acute cerebral injury or stress, such as hypoxic–ischaemic encephalopathy, stroke, and cerebral infection, as well as transient and reversible brain alterations of metabolic or toxic origin; however, congenital brain malformations and genetic disorders are also recognized causes.^7^

Neonatal seizures are associated with an increased risk of neonatal mortality and childhood morbidity, including long‐term neurodevelopmental disorders such as cerebral palsy (CP), intellectual disability, and epilepsy.^8^, ^9^, ^10^, ^11^ However, reported overall cumulative risks of epilepsy later in life after neonatal seizures vary widely from 6% to 56%, and largely dependent on study size and design,^9^, ^12^, ^13^ with few studies being population‐based cohort studies.^1^, ^2^, ^3^, ^4^, ^5^, ^6^ The aetiology of neonatal seizures has a crucial role in determining the risk of epilepsy later in life, with the highest risk observed among children with congenital brain malformations, inborn errors of metabolism, and genetic disorders. While not all children with neonatal seizures develop epilepsy, those who do have their first episode at an earlier age compared to those without seizures. It remains unsettled whether children who do not develop epilepsy in the first years of childhood have a persistently elevated risk later in life. Additionally, whether neonatal seizures are linked to subsequent occurrences of febrile seizures in childhood is unknown.

This register‐based study includes a comprehensive nationwide cohort. We formulated a predetermined analysis plan, hypothesizing that the risk of epilepsy would be significantly heightened in the first 5 years of life among neonatal survivors with neonatal seizures, after which the risk would align with that of neonatal survivors without neonatal seizures. Moreover, we conjectured that the risk of epilepsy in these children would predominantly be aligned with a diagnosis of brain malformation, perinatal brain injury, or a subsequent diagnosis of neurological illness. Finally, we hypothesized that episodes of neonatal seizures would not be associated with an increased risk of febrile seizures in childhood.

## METHOD

### Study design and population

We conducted a register‐based cohort study following all live‐born children born in Denmark between 1st January 1997 and 31st December 2018. We extracted data from four nationwide registries: the Danish Medical Birth Register,^14^ the Danish Register of Causes of Death, the Danish Migration Register, and the Danish National Patient Registry,^15^ linked by the unique Danish personal identification number.^16^

We defined the neonatal period according to gestational age at birth. In children born at term (gestational age ≥ 37 weeks) the neonatal period was the first 28 days after birth. In children born preterm (gestational age < 37 weeks), the neonatal period encompassed the time till the date of term (gestational age = 40 weeks) plus the subsequent 28 days.

We excluded children with missing gestational age and children with a birthweight noted as more than 5SDs from the expected, as this was considered an error related to a wrong gestational age. Furthermore, in alignment with the aims of our study, we thought it more clinically pertinent to examine neonatal survivors; therefore, we excluded all children who died within the neonatal period.

The primary exposure of interest was at least one notation of neonatal seizures (DP90*, where the asterisk signifies that all diagnoses listed in DP90 are included) within the neonatal period in the Danish National Patient Registry, which includes data on all individuals discharged from a Danish public hospital or seen in outpatient clinics. All diagnoses were based on the International Classification of Diseases, 10th Revision.

The primary outcome was epilepsy, defined as at least two notations of either DG40* or DG41* (the asterisk signifies that all diagnoses listed in DG40 or DG41 are included) on two separate dates. We chose at least two notations to reduce the risk of misclassification bias.^17^ The first notation defined the age at onset of epilepsy. In Denmark, the diagnostic criteria for epilepsy follow those of the International League Against Epilepsy (ILAE).^18^ The secondary outcome was febrile seizures, defined as at least one notation of DR560 before the age of 5 years in individuals without a subsequent diagnosis of epilepsy.

To investigate various aetiologies of neonatal seizures and associated comorbidities, we extracted conditions using the International Classification of Diseases, 10th Revision codes outlined in Table S2. Aetiologies were determined by at least one notation in the Danish National Patient Registry before the age of 1 year; however, because of poor registration, an Apgar score below 7 at 5 minutes served as a proxy of perinatal asphyxia.

Preterm birth was categorized as extremely (gestational age < 28 weeks), very (gestational age ≥ 28 and < 32 weeks), and moderately preterm (gestational age ≥ 32 and < 37). Birthweight was categorized into small, appropriate, or large for gestational age, defined as below, between, or above 2S of the expected birthweight.

### Statistical analyses

Descriptive statistics were reported as frequencies with percentages for categorical variables and the mean with SD for continuous variables, which were all symmetrically distributed. We followed all children from their date of birth until outcome, death, emigration, or the 31st December 2018, whichever occurred first. Thus, the maximum follow‐up time was 22 years.

The cumulative risk of developing epilepsy in children with and without neonatal seizures was estimated and illustrated by a plot of the Aalen–Johansen estimator, with death considered as a competing risk. The cumulative risk was estimated according to predefined age intervals (0–1 year, 1–5 years, 5–10 years, 10–15 years, and 15–22 years) and compared using the Gray's test.^19^ The Aalen–Johansen estimator and Gray's test account for the varying lengths of follow‐up as well as competing risks. Children who emigrated without an epilepsy diagnosis were censored at the emigration date. Data management and analyses were performed using R v4.03 (R Foundation for Statistical Computing, Vienna, Austria), with the survival (v3.6–4), cmprsk (v2.2–12), and prodlim (v2023.03.21) packages. Associations between neonatal seizures and childhood onset of epilepsy were analysed using univariable and multivariable Cox regression analyses estimated as cause‐specific hazard ratios (HRs) with 95% confidence intervals (CIs). The multivariable models were adjusted for sex, gestational age, and birth year selected using previous knowledge about causal associations and pathways. In addition, the age at epilepsy onset in each group was described using the median (interquartile range [IQR]); differences between groups were analysed using the Mann–Whitney *U* test.

The impact of various aetiological causes of neonatal seizures on the risk of developing epilepsy was evaluated by categorizing them into three aetiological subgroups and estimating the differences in hazard using multivariable Cox regression analyses, in addition to assessing the cumulative risk. The three aetiology subgroups were: group 1, diagnosis of cerebral infarction, cerebral haemorrhage, cerebral malformation, or kernicterus; group 2, events of perinatal asphyxia (defined by an Apgar score below 7 at 5 minutes) but without a diagnosis of cerebral infarction, cerebral haemorrhage, kernicterus, or cerebral malformation; and group 3 (reference group) defined as those not included in either group 1 or 2. In correlation with the aetiology of seizures, the HR of epilepsy in neonatal survivors with and without neonatal seizures was also stratified according to gestational age with term birth as the reference.

Lastly, the association between neonatal seizures and febrile seizures without subsequent epilepsy was tested similarly.

## RESULTS

A total of 1 330 546 children were born alive in Denmark between 1997 and 2017. We excluded 28 996 (2.2%) children with missing gestational age and 3484 (0.3%) with an outlying birthweight. Lastly, 3689 (0.3%) children who died within the neonatal period were excluded, resulting in a final study cohort of 1 294 377 neonatal survivors being followed for a total of 14.6 million person years (Figure S1). Of these, 1998 (0.2%) had at least one episode of neonatal seizures.

Descriptive differences are presented in Table 1. Neonatal survivors with neonatal seizures were more often born preterm, small for gestational age, large for gestational age, delivered by caesarean section or vacuum extraction, and were born with lower Apgar scores compared to those without neonatal seizures. Furthermore, neonatal survivors with neonatal seizures more frequently had diagnoses of neonatal hypoglycaemia or hyperglycaemia, electrolyte imbalance, sepsis, cerebral infarction, cerebral haemorrhage, or cerebral malformation. Additionally, later neurodevelopmental comorbidities such as CP, intellectual disabilities, attention‐deficit/hyperactivity disorder, and autism spectrum disorder were observed more frequently among neonatal survivors with a prior diagnosis of neonatal seizures. The descriptive characteristics of children who did not survive the neonatal period (*n* = 3689) are presented in Table S1.

**TABLE 1:** Characteristics of children born in 1997–2018 who were alive after the neonatal period.

Variable, *n* (%)	With neonatal seizures *n* = 1998 (0.2%)	Without neonatal seizures *n* = 1 292 379 (99.8%)
Male sex	1115 (55.8)	662 964 (51.3)
Gestational age (weeks), mean (SD)	38.9 (2.8)	39.3 (1.9)
Born extremely preterm (< 28 weeks)	26 (1.3)	2457 (0.2)
Born very preterm (≥ 28 weeks and < 32 weeks)	43 (2.2)	8657 (0.7)
Born moderately preterm (≥ 32 weeks and < 37 weeks)	175 (8.8)	71 645 (5.5)
Born at term (≥ 37 weeks)	1754 (87.8)	1 209 620 (93.6)
Birthweight (g), mean (SD)	3370 (758)	3480 (591)
SGA	124 (6.3)	55 742 (4.3)
AGA	1658 (84.4)	1 174 990 (91.2)
LGA	183 (9.3)	56 979 (4.4)
Caesarean section	747 (37.4)	260 804 (20.2)
Vacuum extraction	327 (16.4)	94 425 (7.3)
Apgar score at 5 minutes <7	409 (21.0)	8084 (0.6)
Hypoglycaemia or hyperglycaemia	398 (19.9)	46 779 (3.6)
Electrolyte imbalance	86 (4.3)	1416 (0.1)
Kernicterus	4 (0.2)	37 (0.003)
Sepsis	368 (18.4)	22 098 (1.7)
Metabolic disorder	11 (0.6)	733 (0.1)
Cerebral infarction	160 (8.1)	98 (0.01)
Cerebral haemorrhage	273 (13.7)	2103 (0.2)
Cerebral malformation	90 (4.5)	2309 (0.2)
CP	407 (20.4)	3968 (0.3)
ADHD	94 (4.7)	36 186 (2.8)
ASD	94 (4.7)	29 906 (2.3)
Intellectual disability	56 (2.8)	7369 (0.6)
Chromosomal anomalies	41 (2.1)	3709 (0.3)

*Note*: Missing values (*n* [%]): birthweight: 4701 (0.4); Apgar score at 5 minutes: 10 674 (0.8). Abbreviations: ADHD, attention‐deficit/hyperactivity disorder; AGA, appropriate for gestational age; ASD, autism spectrum disorder; CP, cerebral palsy; LGA, large for gestational age; SGA, small for gestational age.

**FIGURE 1:**
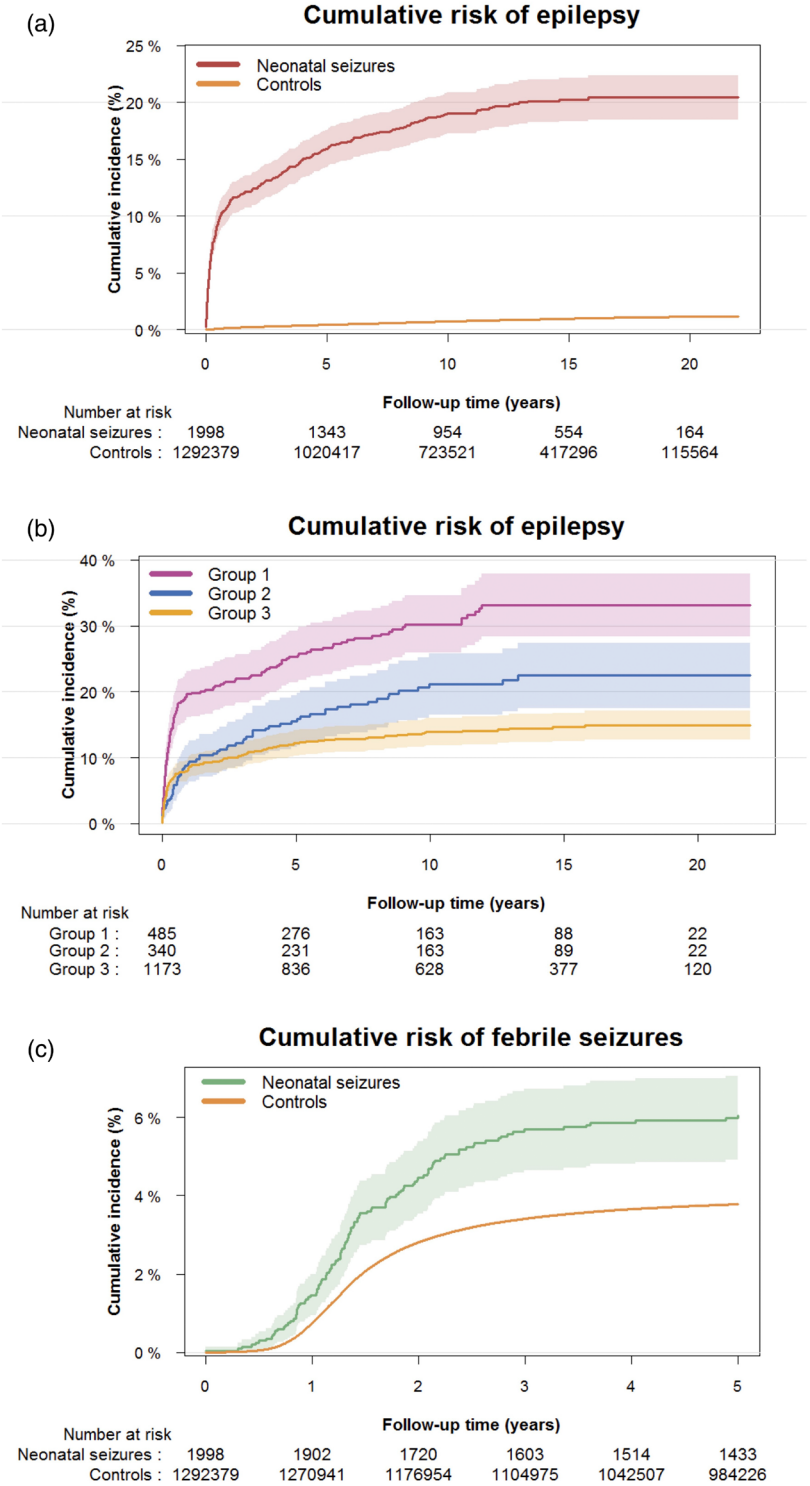
(a) Cumulative risk of epilepsy based on the Aalen–Johansen estimator in children with and without neonatal seizures. (b) Cumulative risk of epilepsy based on the Aalen–Johansen estimator in children with neonatal seizures stratified according to the aetiology of neonatal seizures; group 1, diagnosis of cerebral infarction, cerebral haemorrhage, kernicterus, or cerebral malformation; group 2, events of intrapartum asphyxia (defined by an Apgar score below 7 at 5 minutes) and not included in group 1; and group 3 (reference group), unknown aetiology defined as those not included in either group 1 or group 2. (c) Cumulative risk of febrile seizures without subsequent epilepsy in children with and without neonatal seizures.

Among survivors with neonatal seizures *n* = 367 of 1998 (18.4%) were diagnosed with epilepsy compared to *n* = 9619 of 1 292 379 (0.7%) among survivors without neonatal seizures (Table 2). When considering death as a competing risk, the overall cumulative incidence of epilepsy within 22 years of follow‐up for neonatal survivors with neonatal seizures was 20.4% (95% CI = 18.5–22.3) compared to 1.15% (95% CI = 1.12–1.18) among survivors without seizures (*p* < 0.001) (Table 2 and Figure 1a). Thus, neonatal survivors with seizures had a 17.7 (95% CI = 16.5–18.9) times higher cumulative risk of epilepsy. Furthermore, the adjusted HR for epilepsy was 27.11 (95% CI = 24.42–30.09) in survivors with seizures (Table 2).

**TABLE 2:** Cumulative incidence and hazard ratio of epilepsy among children with and without neonatal seizures.

	Children with neonatal seizures (*n* = 1998)			Children without neonatal seizures (*n* = 1 292 379)			*p*
Age (years)	*n*	Number of new cases with epilepsy	Cumulative incidence, % (95% CI)^a^	*n*	Number of new cases with epilepsy	Cumulative incidence, % (95% CI)^a^	
0–1	1998	228	11.4 (10.0–12.8)	1 292 379	1976	0.15 (0.15–0.16)	< 0.001^b^
1–5	1726	81	15.9 (14.3–17.6)	1 279 193	3173	0.43 (0.42–0.44)	< 0.001^b^
5–10	1343	45	19.0 (17.2–20.9)	1 020 417	2561	0.72 (0.70–0.74)	< 0.001^b^
10–15	954	12	20.3 (18.3–22.2)	723 521	1398	0.96 (0.94–0.98)	< 0.001^b^
15–22	554	<5	20.4 (18.5–22.3)	417 296	511	1.15 (1.12–1.18)	< 0.001^b^
Crude HR (95% CI)	28.40 (25.59–31.52)			Ref.			< 0.001^c^
Adjusted HR (95% CI)^d^	27.11 (24.42–30.09)			Ref.			< 0.001^c^

Abbreviations: CI, confidence interval; HR, hazard ratio; Ref., reference.

^a^ Aalen–Johansen estimator.

^b^ Gray's test.

^c^ Cox regression.

^d^ Adjusted for sex, gestational age, and birth year.

Categorized according to age at diagnosis, it showed that 11.4% of neonatal survivors with seizures received their epilepsy diagnosis within the first year of life. Considering death as a competing risk, an additional 4.5% (15.9% minus 11.4%) were diagnosed between the ages of 1 year and 5 years, 3.1% (19.0% minus 15.9%) within 5 to 10 years, and 1.4% (20.4% minus 19.0%) between 10 years and 22 years (Table 2). At all age intervals, the risk of epilepsy was significantly increased compared to neonatal survivors without neonatal seizures. The median age at diagnosis of epilepsy among neonatal survivors with neonatal seizures was 5 months (IQR = 1 month–3 years 2 months) compared to 4 years 6 months (IQR = 1 years 5 months–8 years 11 months) in survivors without neonatal seizures (*p* < 0.001).

When analysing the aetiology of neonatal seizures, we found that the adjusted HRs of epilepsy were significantly higher among neonatal survivors diagnosed with cerebral infarction, cerebral haemorrhage, kernicterus, or cerebral malformation (structural brain damage) and in those with asphyxia without the aforementioned diagnoses compared to neonatal survivors with seizures of other or unknown aetiology (2.49 [95% CI = 1.98–3.14] and 1.49 [95% CI = 1.12–1.98]). Similarly, the cumulative risk of epilepsy was also significantly higher in the two subgroups at 33.1% (95% CI = 28.4–37.9) and 22.4% (95% CI = 17.5–27.4) respectively versus 14.9% (95% CI = 12.7–17.1) in the reference group (*p* < 0.001) (Table 3 and Figure 1b).

**TABLE 3:** Relative risk of epilepsy among children with neonatal seizures stratified according to aetiology.

	Neonatal seizures and cerebral infarction, haemorrhage, malformation, or kernicterus (*n* = 485 [24.3])	Neonatal seizures and birth asphyxia only (*n* = 340 [17.0])	Neonatal seizures without cerebral infarction, haemorrhage, malformation, kernicterus, or asphyxia (*n* = 1173 [58.7])
Epilepsy, *n* (%)	141 (29.1)	67 (19.7)	159 (13.6)
Crude HR (95% CI)	2.44 (1.95–3.07)	1.48 (1.11–1.97)	Ref.
Adjusted HR (95% CI)^a^	2.49 (1.98–3.14)	1.49 (1.12–1.98)	Ref.

Abbreviations: CI, confidence interval; HR, hazard ratio; Ref., reference.

^a^ Adjusted for sex, birth year, and gestational age.

When stratifying according to gestational age (preterm vs term), in the multivariable Cox regression models, the adjusted HR of epilepsy after neonatal seizures was 1.59 (95% CI = 1.49–1.70) times higher in neonatal survivors born preterm compared to survivors born at term.

The cumulative incidence of febrile seizures without subsequent epilepsy was significantly higher in neonatal survivors with neonatal seizures compared to survivors without neonatal seizures when considering death as a competing risk (6.2% [95% CI = 5.1–7.3] vs. 3.8% [95% CI = 3.7–3.8], *p* < 0.001) (Figure 1c), with an adjusted HR of 1.61 (95% CI = 1.35–1.94).

## DISCUSSION

In this large population‐based cohort study, we confirmed a substantially increased risk of epilepsy after neonatal seizures, with approximately one in five neonatal survivors affected. The risk of epilepsy was highest in the first year of life but remained significantly elevated throughout childhood and adolescence. Furthermore, the risk of epilepsy after neonatal seizures was highest among those with cerebral malformation or perinatal brain injury, although children with perinatal asphyxia alone also had an increased risk compared to other aetiologies. Additionally, the risk of febrile seizures was also significantly higher in neonatal survivors with neonatal seizures, although not to the same extent as the risk for epilepsy.

To our knowledge, this is, to date, the most extensive population‐based study examining the incidence of epilepsy after neonatal seizures, confirming findings from more recent European studies.^1^, ^20^ More than half of the children in our study were diagnosed with epilepsy before the age of 1 year, supporting that epilepsy is diagnosed at a much younger age among neonatal survivors with a history of neonatal seizures.^1^, ^20^, ^21^ Interestingly, we found that the increased risk of epilepsy persisted throughout childhood and adolescence. Although the difference in risk decreased with age, the latent period from neonatal seizures to the manifestation of epilepsy may be longer in some individuals than previously reported. However, the pathophysiology linking neonatal seizures to earlier manifestations of epilepsy is poorly understood.^4^, ^22^, ^23^, ^24^, ^25^

Aetiologies of epilepsy after neonatal seizures partly overlap with the aetiologies of neonatal seizures.^21^, ^26^ Children with neonatal seizures due to cerebral infarction, cerebral haemorrhage, cerebral malformations, or kernicterus were at the greatest risk of later epilepsy, probably reflecting more extensive structural cerebral impairment. The proportion of children with brain abnormalities was lower in our study compared to more contemporary studies,^4^ most probably because magnetic resonance imaging (MRI) was not used routinely in Denmark in children with neonatal seizures during the early part of the study period.

We used the Apgar score as a proxy for perinatal asphyxia, choosing a cut‐off of 7 after 5 minutes to include both moderate and severe asphyxia, and found a higher risk of epilepsy among these children compared to those with neonatal seizures of other aetiologies. Although discrepancies exist regarding hypoxic–ischaemic encephalopathy as a risk factor for later epilepsy, this may relate to the severity of hypoxic–ischaemic encephalopathy.^5^, ^13^, ^27^ We also found a small but increased risk of epilepsy after neonatal seizures in children born preterm compared to children born at term. Limited data with conflicting results exist on the association between gestational age and epilepsy after neonatal seizures.^28^ Our results suggest that neonatal seizures are more harmful to the preterm brain, but the higher risk may also relate to differences in the aetiologies of neonatal seizures between children born preterm and at term. Furthermore, therapeutic hypothermia in children born at term with severe hypoxic–ischaemic encephalopathy reduces the risk of neurological disability and neonatal seizures. However, how this influences the relationship between neonatal seizures and epilepsy is speculative.

The purpose of more extensive early diagnostics regarding the aetiology of neonatal seizures (imaging or genetics) is to identify the relatively few infants for whom early intervention and precision therapy are available, such as infantile spasms in children with tuberous sclerosis. In addition, early diagnostics may reassure the parents of infants with normal test results about their child's better prognosis. However, this approach may also have downsides, including unnecessarily alarming parents of infants who will do well^29^ and potentially labelling the child as vulnerable, leading to overprotection.^30^ Clinically, MRI and genetic testing should be considered alongside the medical history, while from a research perspective, knowledge of genetic traits in epilepsy after neonatal seizures may improve our understanding of the underlying pathophysiology. We find that most paediatric neurologists would agree that understanding the basis of such an important condition is crucial for diagnostic accuracy, treatment planning, and providing parents with a more comprehensive understanding of their child's potential future.

Disentangling the effect of the pathology behind neonatal seizures and the neonatal seizures themselves is challenging. Our data demonstrate that this association extends into adolescence, rendering this question even more pertinent. Not all children with structural brain injury or abnormalities and neonatal seizures develop epilepsy; thus, some children are more susceptible. More subtle brain injury or genetically determined variability in seizure thresholds may be in play. As speculated by Pisani et al.,^25^ a synergistic effect between the underlying brain disorder and neonatal seizures is possible. Evidence suggests that neonatal seizures may alter neuronal plasticity and excitability, potentially through ongoing inflammation.^31^, ^32^, ^33^ Interestingly, a more significant proportion of pathogenic variants in epilepsy genes has been found among children with epilepsy after acute symptomatic neonatal seizures.^33^ Overall, it is thus possible that neonatal seizures are a symptom of acute brain injury leading to secondary epileptogenesis or that neonatal seizures occur in children who are genetically predisposed to epilepsy or febrile seizures.

Other neurodevelopmental disorders (genetic syndromes, CP, intellectual disability, neuropsychiatric disorders) often coexist with epilepsy, which may be explained by an inherent genetic risk. However, a causal relationship between later epilepsy and comorbidities was not possible to disentangle statistically from the current study. Few studies report on the relationship between neonatal seizures and later febrile seizures without subsequent epilepsy.^5^ Whether the increased risk of febrile seizures after neonatal seizures can be explained by a lower seizure threshold due to perinatal factors, genetic factors, or neonatal seizures per se is not clear. A genetic predisposition to febrile seizures is well known, and some genetic variants may predispose to both neonatal and febrile seizures.^34^

We present data from a large population‐based study, the largest to date, with nearly 2000 cases of neonatal seizures, which is a major strength of our study along with the longest follow‐up to date. Another strength are the Danish registries linked to the unique Danish personal identification number, which enable these large studies. We interpret our results as valid and generalizable to populations with similar demographics. However, our study also has limitations. In the clinical handling of seizures in neonates, the clinician uses the ILAE neonatal seizure definition.^7^ For the later diagnosis of epilepsy, the ILAE classification of epilepsy is applied.^18^ In the classification of epilepsy, diagnosis must follow either two unprovoked seizures at least 24 hours apart, one unprovoked seizure and a risk above 60% for recurrence, or classification of an epilepsy syndrome. In neonates, the clinical diagnosis of neonatal seizures can be difficult because there can be many epileptic and non‐epileptic clinical manifestations in often clinically ill neonates. The neonatal classification emphasizes the role of the electroencephalogram (EEG) in the diagnosis of seizures. Many neonatal seizures are electrographic‐only, with no evident clinical features. Clinical events without an EEG correlate are not included. Our set‐up depends on routine diagnosis and reporting from hospital admissions; a potential bias is an underestimation of the true number of neonatal seizures if electrographic‐only seizures or milder manifestations are not registered. Such bias would contribute to a higher risk of epilepsy in our group of interest because only severe cases are noted with neonatal seizures. In contrast, an overestimation of neonatal seizures is also possible; however, since the prevalence of neonatal seizures in our cohort was low compared with previous population‐based studies, we find this less likely. Diagnosis of neonatal seizures with the availability of amplitude‐integrated EEG, EEG, and neurophysiologist assistance has evolved; although EEG monitoring with video is the criterion standard to diagnose neonatal seizures, this may never be implemented systematically in clinical care in all neonatal care units. Our study was based on population‐based data that rely on reporting from vital statistics and hospital discharge diagnoses. All neonates were included, both from low‐acuity and high‐acuity centres. EEG and amplitude‐integrated EEG has been available in many centres in the period, nevertheless it is likely that the diagnoses may, in many cases, reflect a clinical diagnosis of seizures.

A misclassification of neonatal onset epilepsy or epileptic encephalopathy may also have biased our results towards a greater incidence of epilepsy in the first year of life. However, we consider the magnitude of this bias to be of minor influence because early‐onset epilepsy is rare. We used the Apgar score as a proxy of perinatal asphyxia, which could introduce a bias towards a lower risk of epilepsy as some of the children may have been given a low Apgar score of other causes than asphyxia.

In our study, we have relied on register‐based epilepsy diagnoses. This will include some cases that do not fulfil the criteria for epilepsy, but we have sought to increase specificity by including only cases that had two epilepsy diagnoses. Danish clinicians are in general encouraged to follow the ILAE classification of epilepsies; however, in our study, we were not able to confirm if in fact the diagnoses were in accordance with the ILAE criteria. In a study with clinical validation of register diagnoses, a false discovery rate of 29% was reported.^35^ Another approach that could have been applied (but was not available to us) is diagnosis confirmation using data mining, where a lower positive predictive value was found, but this method does not include information from EEG, MRI, antiseizure medication prescription, or number of International Classification of Diseases, 10th Revision diagnoses.^36^

Lastly, we cannot exclude that some of the children born in the most recent years and diagnosed with febrile seizures may develop epilepsy at an older age and thus be misclassified, but we find this potential bias of minor significance to our results.^37^

### Conclusions

Our study confirms a substantial risk of epilepsy after neonatal seizures in neonatal survivors, with the highest risk observed in the first year of life but persisting into adolescence. Structural brain abnormalities were associated with the greatest risk of epilepsy. Furthermore, the risk of febrile seizures was also increased after neonatal seizures, although to a much lesser extent. Importantly, four out of five neonatal survivors with a history of neonatal seizures did not develop epilepsy. Future studies should explore the potential for genetic predisposition to identify children at particular risk, enabling tailored follow‐up and preventive measures.

## CONFLICT OF INTEREST STATEMENT

The authors have stated that they had no interests which might be perceived as posing a conflict or bias.

## Supporting information


**Table S1:** Characteristics of the children who died within the neonatal period.

**Table S2:** ICD‐10 codes.

**Figure S1:** Flow chart of the cohort.

## Data Availability

Data is available on request to the first author of the manuscript.
