# Supplementary material for: The risk of epilepsy after neonatal seizures
Source: Dev Med Child Neurol. 2025 Feb 19;67(9):1157–64. doi: 10.1111/dmcn.16255 (PMC12336401; doi:10.1111/dmcn.16255)
Supplement: Supplementary file 1 — Table S1: Characteristics of the children who died within the neonatal period. [file DMCN-67-1157-s003.docx]

**Supplementary Table 1.** Characteristics of the children who died within the neonatal period

| **Variables,** n (%) | **Died in the neonatal period**  n=3846 | |
| --- | --- | --- |
|  | **With neonatal seizures**  n=195 (5.1%) | **Without neonatal seizures**  n=3651 (94.9%) |
| Male sex | 109 (55.9) | 2022 (55.4) |
| Gestational age (weeks), mean (SD) | 36.2 (4.92) | 29.1 (6.86) |
| Extremely premature (<28 weeks) | 19 (9.7) | 1953 (53.5) |
| Very premature (28–31 weeks) | 16 (8.2) | 373 (10.2) |
| Moderately premature (32–36 weeks) | 31 (15.9) | 492 (13.5) |
| Born at term (>36 weeks) | 129 (66.2) | 833 (22.8) |
| Birth weight (grams), mean (SD) | 2820 (1110) | 1520 (1190) |
| SGA | 29 (15.6) | 830 (23.8) |
| AGA | 146 (78.5) | 2512 (71.9) |
| LGA | 11 (5.9) | 151 (4.3) |
| Cesarean section | 111 (56.9) | 1413 (38.7) |
| Vacuum extraction | 29 (14.9) | 110 (3.0) |
| Apgar score 5min, 4–7 | 60 (32.8) | 599 (19.3) |
| Apgar score 5min, 0-3 | 51 (27.9) | 1262 (40.6) |
| Hypo/hyperglycemia | 48 (24.6) | 325 (8.9) |
| Electrolyte imbalance | 10 (5.1) | 45 (1.2) |
| Kernicterus | 0 | 0 |
| Sepsis | 40 (20.5) | 433 (11.9) |
| Metabolic disorders | 6 (3.1) | 13 (0.4) |
| Cerebral infarction | $\leq$5 ($\leq$ 2.6) | $\leq$5 ($\leq$ 0.1) |
| Cerebral haemorrhage | 43 (22.1) | 428 (11.7) |
| Cerebral malformation | 9 (4.6) | 150 (4.1) |
| Abbreviations: n, number; SD, standard deviation; SGA, small for gestational age; AGA, appropriate for gestational age; LGA, large for gestational age. Missing values for children without neonatal seizures who died in the neonatal period: birth weight 158 (4.3), asphyxia 5min: 543 (14.9). Missing values for children with neonatal seizures, who died in the neonatal period, n (%): birth weight 9 (4.6), asphyxia 5min: 12 (6.2). | | |
