# Supplementary material for: The risk of epilepsy after neonatal seizures
Source: Dev Med Child Neurol. 2025 Feb 19;67(9):1157–64. doi: 10.1111/dmcn.16255 (PMC12336401; doi:10.1111/dmcn.16255)
Supplement: Supplementary file 2 — Table S2: ICD‐10 codes. [file DMCN-67-1157-s002.docx]

**Supplementary Table 2** ICD-10 codes

|  | ICD-10 codes |
| --- | --- |
| Exposure  Neonatal seizures | DP90* |
| Outcome  Epilepsy  Febrile seizure | DG40*-41*  DR560* |
| Etiology neonatal seizures  cerebral infarction  cerebral hemorrhage  hypo/hyperglycemia  electrolyte imbalance  kernicterus  sepsis  metabolic disorders  cerebral malformation | DI63*  DI61*–DI62*, DP101, DP52*, DP548, DP549, DR58, , DS064*–DS066*, DS068B, DS068D  DE15*, DE160, DE161, DE161B, DE162, DP70*  DE834B; DE835D, DE871, DE871A, DP71*, DP742*  DP57*  DA267, DA40*, DA41*, DA427, DA499A, DA548G, DB377, DP36*, DT802D1, DT814D  DE70*–DE74*, DE78*  DG282, DQ00*–DQ07*, DQ273A, DQ283, DQ283E, DQ892G |
| Comorbidities  cerebral palsy  ADHD^a^  ASD^b^  intellectual disabilities  chromosome abnormalities | DG80*  DF90*  DF84*  DF70*–DF79*  DF842, DQ851, DQ90*–DQ93*, DQ95*–DQ99* |
