# Supplementary figures and images for: The risk of epilepsy after neonatal seizures
Source: Dev Med Child Neurol. 2025 Feb 19;67(9):1157–64. doi: 10.1111/dmcn.16255 (PMC12336401; doi:10.1111/dmcn.16255)

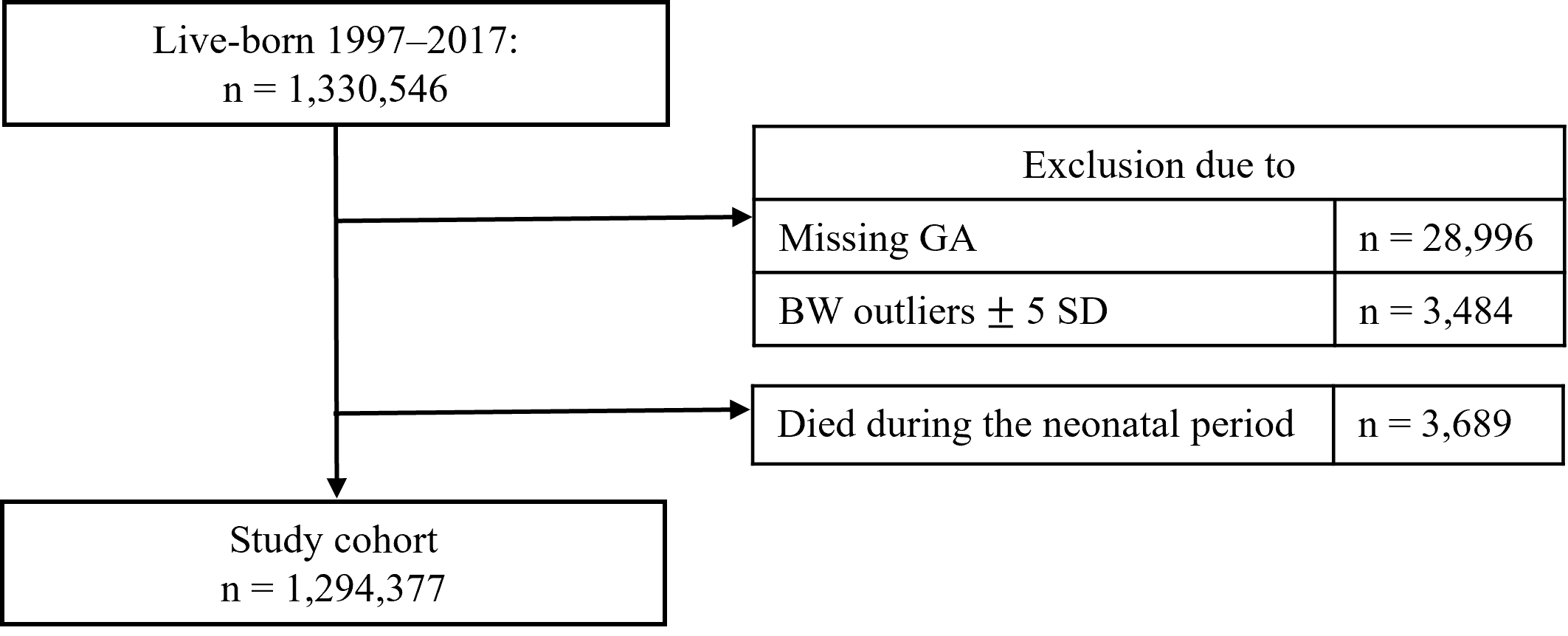

Supplement: Supplementary file 3 — Figure S1: Flow chart of the cohort. [file DMCN-67-1157-s001.tif]
